# Social and Poor Law Problems

**Published:** 1907-03-02

**Authors:** 


### SOCIAL AND POOR LAW PROBLEMS.

### AN EXPERIMENT IN MILK SUPPLY.

Yorkshire is taking hold of the problem of supplying pure milk, and the result of the first year's working of the Wensleydale Pure Milk Society is such as to show what can be done by an Association which, while it does not despise reasonable profit-making, has as its first aim the improving of the food supply of the neighbourhood in an important particular. The Society's motto might be taken from a dictum of one of the directors, Mr. Philip Burtt, who defined their aim as being "philanthropy on a commercial basis."

Commercially, the Society has as yet to admit a loss, but this is a not infrequent feature of the first year's working of any company, and, as the Wensleydale Society commenced operations only in October 1905, it has only one year's complete working to show, and, though that has to confess to a loss of £773 12s. 9d., the prospects of the company are good, and the succeeding quarter shows a satisfactory profit. A bottling factory is established at Northallerton, where, by June 30 of last year, the amount bottled daily was 250 gallons. Distributing centres have been established at Scarborough and at Leeds, where the setting up of a second centre is in contemplation. Besides these special depots belonging to the society, the milk bottled by it is being sold at Newcastle-on-Tyne, West Hartlepool, Middlesbrough, Wallsend, South Shields, Hebburn, Jarrow, and Murton Colliery. The Society expresses special appreciation of the help given by "the active enterprise of the Newcastle-on-Tyne Co-operative Society." As the co-operative societies have their shareholders for the most part among the working class, this acknowledgment shows that the distribution of pure milk is going on actively among that part of the population who most need protection in this important matter.

But good distribution is of little value without good production. If the farms whence the milk is procured are bad it avails little that the shops where it is sold are good, though perhaps it may save expense in sterilisation. But pure milk, which needs no treatment, is better far than milk in which benign germs are destroyed in the process of getting rid of dangerous ones. Therefore, we think that one of the most important parts of the Wensleydale Society's work is the improvement of conditions in the farms where the milk is produced. The report states that upon eight farms new dairies have been built, and upon eleven farms an alteration of buildings has been made in order to comply with the Society's regulations as to filtering and cooling the milk. Thirteen new milk-cooling plants, and twenty-four special milk filters have been erected. Three new cow-houses have been built by the landlords at the instigation of the Society, and many others improved as regards floors, drainage, ventilation, and light.

This, though it does not show in the balance-sheet, is really one of the most important departments of the Society's work. When farmers realise that having their cowsheds and dairies in a sanitary condition secures them a sale for their milk, they will be ready to fall in with the suggestions of the Society on these matters. Of course, the conversion of producers depends largely on the conversion of consumers. If the Society could show next year a table of the infant mortality among families using their milk as compared with that of an equal number of families who are not so fastidious, they would do a great deal towards proving the importance of cleanliness in milk supply, and this would help to make the Society as satisfactory as a commercial as it already is as a philanthropic undertaking.

